# Systemic Involvement of Leukocytoclastic Vasculitis in Skin of Color

**DOI:** 10.7759/cureus.82105

**Published:** 2025-04-11

**Authors:** Mana Nasseri, Justin L Jia, James Croffoot

**Affiliations:** 1 Department of Dermatology, George Washington University School of Medicine and Health Sciences, Washington, D.C., USA; 2 Department of Dermatology, George Washington University, Washington, D.C., USA; 3 Department of Internal Medicine, Veteran Affairs Medical Center, Washington, D.C., USA; 4 Department of Internal Medicine, George Washington University, Washington, D.C., USA

**Keywords:** leukocytoclastic vasculitis (lcv), multisystem involvement, parainfluenza pneumonia, skin of color, small vessel vasculitis

## Abstract

Leukocytoclastic vasculitis (LCV) is an immune-mediated small-vessel vasculitis with various underlying causes. We present a unique case of LCV in an elderly man with Fitzpatrick phototype V skin to highlight multi-organ signs associated with this rare pathology. Our patient presented with an evolving, diffusely spread rash following respiratory symptoms accompanied by muscle weakness, scant hemoptysis, and decreased visual acuity. A comprehensive diagnostic approach, including skin biopsy, revealed LCV with associated eosinophilia. Treatment through a multidisciplinary approach included systemic steroids, topical ointments, and empiric antibiotics. The patient showed gradual improvement and was discharged after nine days of hospitalization. Differential diagnoses for the rash included infections, inflammatory processes, and autoimmune vasculitides such as immunoglobulin A (IgA) vasculitis, urticarial vasculitis, cryoglobulinemic vasculitis, and anti-neutrophil cytoplasmic antibody (ANCA)-associated vasculitides. While most instances of LCV are typically skin-limited and self-resolving, this case highlights diffuse systemic involvement requiring a broad diagnostic perspective.

## Introduction

Leukocytoclastic vasculitis (LCV) often presents as palpable purpura and involves small-caliber venules in the skin [1]. Although frequently idiopathic, LCV can be triggered by infections, medications, malignancies, autoimmune connective tissue disorders, or inflammatory bowel diseases [1,2]. Biopsy of these lesions is diagnostic, and histopathology typically reveals a neutrophilic infiltrate surrounding the vessel wall, and endothelial cell damage, with or without fibrinoid necrosis [3]. Variations in the inflammatory infiltrate, including lymphocytes or eosinophils, may suggest alternate overlapping diagnoses such as drug-induced LCV [4]. LCV is often described as a single-organ vasculitis limited to the skin; however, extracutaneous manifestations can occur in 30% of cases, involving organs such as the kidneys, lungs, and eyes, and may necessitate a broader diagnostic and management approach [1,5]. Clinically, it can resemble other small-vessel vasculitides, including immunoglobulin A (IgA) vasculitis or antineutrophil cytoplasmic antibody (ANCA)-associated vasculitis, complicating the diagnostic process.

In patients with richly pigmented skin, LCV may be particularly difficult to recognize. While LCV is more commonly described in lighter skin phototypes, its features may appear differently in darker skin, where erythema can be violaceous or subtle, and purpura less readily appreciated. The interplay between inflammation and melanin density can obscure classic morphologies and contribute to diagnostic delays [6]. Limited representation of richly pigmented skin in dermatologic literature and training resources may further contribute to underrecognition and delayed care. Increasing awareness of these pigment-related differences is essential for equitable and timely diagnosis [7].

We present a unique case of LCV with eosinophilia in an elderly man with Fitzpatrick phototype V skin, likely triggered by Parainfluenza pneumonia in the setting of recent high-dose corticosteroid use. This case highlights the challenges of diagnosing LCV in skin of color and emphasizes the importance of histopathologic confirmation and multidisciplinary care. By improving recognition of atypical LCV presentations across diverse skin tones, clinicians can help reduce diagnostic delays and address health disparities in dermatologic care. 

## Case presentation

We describe a 70-year-old man with a medical history of focal segmental glomerulosclerosis (FSGS), hypertension, and a distant history of colon cancer presenting with one week of productive cough, scant hemoptysis, subjective fever, and shortness of breath. Notably, the patient was diagnosed with FSGS and started prednisone 60 mg daily one month before admission, but discontinued it after two weeks due to the emergence of his newfound respiratory symptoms. Over the following two weeks, he developed a diffusely evolving rash starting on the lower extremities, progressively spreading cephalically. While the rash was mostly in the same stage, new lesions appeared as the initial rash began to improve and were accompanied by myalgia, swelling of the tongue and eyes, leading to decreased visual acuity, and abdominal pain.

**Figure 1:**
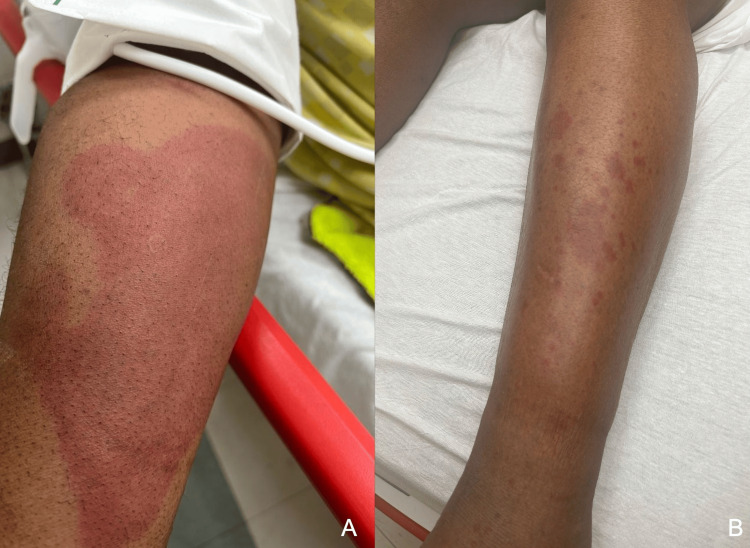
Erythematous to violaceous non-blanching macules and papules coalescing into thin plaques on the (A) upper and (B) lower extremities.

**Figure 2:**
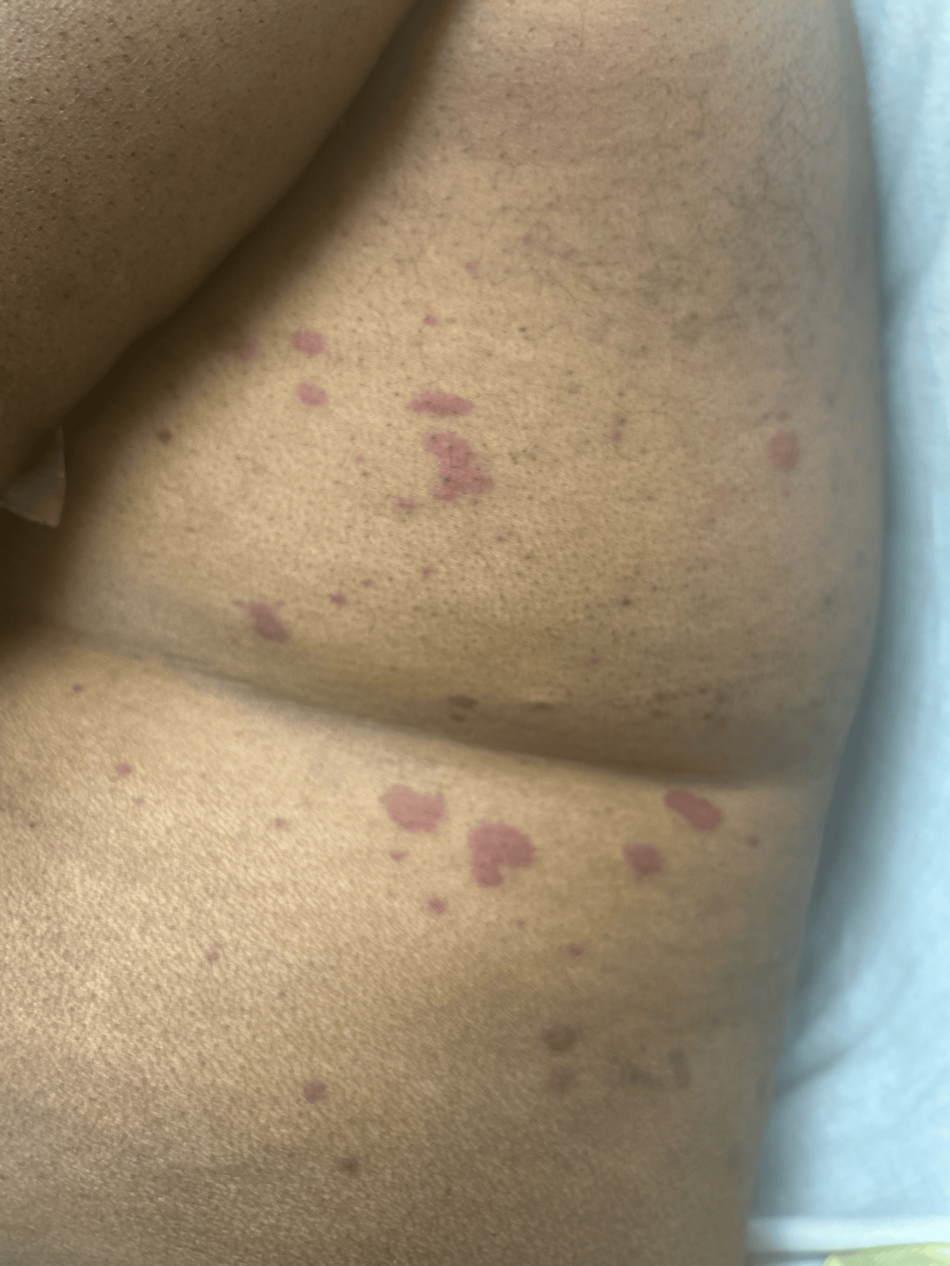
Well-defined salmon and violaceous macules on the trunk.

On admission, he was tachycardic but otherwise hemodynamically stable and afebrile. He had prominent, round, erythematous-violaceous macules coalescing into large patches distributed throughout his legs, groin, trunk, back, and arms (Figures 1-2). The patient also had reddish-purple edematous plaques in the perioral region and buccal mucosa. These palpable lesions were non-blanching, warm to touch, and presented with various stages of healing from raised to flat, consistent with LCV. There was no lymphadenopathy, pain, or pruritus associated with his skin eruption. Auscultation revealed rhonchi most prominent in the bilateral upper lung fields. Furthermore, his case was complicated by diffuse muscle weakness, right greater than left eye chemosis and conjunctivitis causing vision impediment, and scant hematuria with nephrotic range proteinuria in the setting of known primary FSGS of unclear etiology.

**Figure 3:**
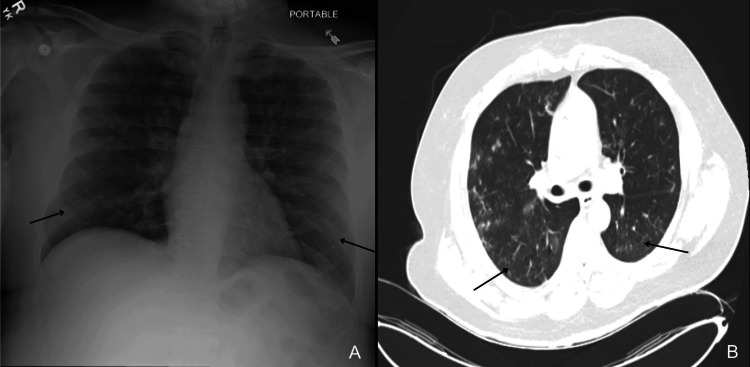
Anteroposterior (AP) chest X-ray (A) demonstrating bilateral pulmonary infiltrates and axial chest computed tomography (CT) scan (B) showing bilateral ground-glass opacities.

Further workup included chest x-ray and computed tomography (CT) scan, which revealed diffuse infiltrates, suggestive of pneumonia (Figure 3). Due to recent steroid use, *Pneumocystis jirovecii *pneumonia (PCP) and aspergillosis were considered. His bloodwork was notable for transaminitis (aspartate aminotransferase, 104 U/L, alanine aminotransferase, 65 U/L) and elevated inflammatory markers C-reactive protein (CRP), consistent with systemic inflammation. There was no evidence of leukocytosis or peripheral eosinophilia, which initially made drug-induced vasculitis less likely. His renal function was near recent baseline, with elevated blood urea nitrogen (BUN) and creatinine levels (BUN 38 mg/dL, creatinine 2.57 mg/dL), consistent with his known kidney disease (Table 1). The initial infectious workup, employing a comprehensive molecular diagnostic test capable of detecting a broad range of bacteria and viruses, identified a positive result for the presence of the Parainfluenza virus, with all other tested pathogens returning negative results.

**Table 1:** Laboratory results on admission, including inflammatory markers, renal function, and hematologic parameters. Abnormal values are denoted as follows: (H) = high, (L) = low. Abbreviations: CRP = C-reactive protein; CO₂ = carbon dioxide; PO₄ = phosphate; ALP = alkaline phosphatase; AST = aspartate aminotransferase; BUN = blood urea nitrogen; ALT = alanine aminotransferase; WBC = white blood cells; RBC = red blood cells; HGB = hemoglobin; HCT = hematocrit; MCV = mean corpuscular volume; MCH = mean corpuscular hemoglobin; MCHC = mean corpuscular hemoglobin concentration; RDW = red cell distribution width; PLT = platelets; MPV = mean platelet volume

Test Name	Result	Units	Reference Range
Sodium	138	mmol/L	135 - 147
CRP	13.1 (H)	mg/dL	0.00 - 0.70
Potassium	4.9	mmol/L	3.5 - 5.3
Chloride	103	mmol/L	100 - 109
CO_2_	28	mmol/L	21 - 31
Calcium	7.7 (L)	mg/dL	8.9 - 10.5
PO_4_	4.1	mg/dL	2.5 - 4.5
Magnesium	1.6	mg/dL	1.6 - 2.4
Total protein	4 (L)	g/dL	6.0 - 8.2
Albumin	2 (L)	g/dL	3.7 - 5.0
ALP	76	U/L	43 - 130
AST	104 (H)	U/L	8 - 40
ALT	65 (H)	U/L	6 - 33
Total bilirubin	0.4	mg/dL	0.2 - 1.2
Direct bilirubin	<0.2	mg/dL	0.0 - 0.2
Glucose	127	mg/dL	70 - 121
BUN	38 (H)	mg/dL	6 - 23
Creatinine, serum	2.57 (H)	mg/dL	0.7 - 1.5
Anion gap	7 (L)	mmol/L	8 - 18
Lactic Acid	2.5	mmol/L	0.6 - 2.2
WBC	5.8	K/cmm	3.2 - 9.5
RBC	3.8 (L)	Mil/cmm	4.21 - 5.84
HGB	11.6 (L)	g/dL	13.2 - 17.3
HCT	34.6 (L)	%	38.6 - 50.2
MCV	90.5	fL	80.2 - 96.7
MCH	30.3	pg	26.6 - 33.9
MCHC	33.4	%	32.8 - 35.7
RDW	14.9 (H)	%	10.7 - 14.1
PLT	204	K/cmm	152 - 375
MPV	8.4	fL	6.4 - 10.4
Neutrophils (%)	73.5	%	38.1 - 75
Lymphocytes (%)	20.1	%	11.9 - 43.1
Monocytes (%)	5.7	%	4.1 - 12.9
Eosinophils (%)	0.4	%	0.0 - 4.8
Basophils (%)	0.3	%	0.0 - 1.65
Neutrophils (Absolute)	4.3	K/cmm	1.3 - 6.2
Lymphocytes (Absolute)	1.2	K/cmm	0.8 - 3.1
Monocytes (Absolute)	0.3	K/cmm	0.13 - 0.83
Eosinophils (Absolute)	0	K/cmm	0.0 - 0.33
Basophils (Absolute)	0	K/cmm	0.0 - 0.1

The patient was started on prednisone 30 mg daily and triamcinolone 0.1% ointment twice daily. Per infectious disease recommendations, the patient started ceftriaxone for one day, then switched to cefdinir, azithromycin, and atovaquone as PCP prophylaxis. The following day, the patient was noted to have persistent chemosis (greater on the right than left), conjunctivitis, and eyelid swelling. Ophthalmologic exam showed no scleritis or intraocular involvement, and a brain/orbit MRI was unremarkable. The preliminary results of a skin biopsy performed on admission revealed leukocytoclastic vasculitis with profound eosinophilia. By the third day of admission, the patient showed overall improvement, leading to discontinuation of prednisone due to rapid clinical recovery, concern for potential side effects, and low suspicion for an autoimmune etiology. On the fifth day, the patient developed herpes labialis, treated with valacyclovir, but continued to otherwise improve until the next day when new large and indurated erythematous plaques appeared extending over his right lateral thigh, slowly resolving after escalation from triamcinolone to clobetasol 0.05% ointment. Over the following week, the patient's respiratory symptoms and generalized myalgia resolved, his ophthalmic symptoms improved without intervention, and his rash and swelling continued to lessen without signs of remission. The final culture results from a bronchoscopy conducted on the second day of admission revealed no fungal or bacterial growth, and the bronchioalveolar lavage sample did not recover any malignant cells on pathology. Further workup for rheumatologic causes, including tests for rheumatoid factor, antineutrophil cytoplasmic antibodies, cryoglobulins, anti-cyclic citrullinated peptide antibodies, serum complements, and antinuclear antibodies, yielded unremarkable results. By Day 9, the patient completed a course of empiric antibiotics and was discharged home with instructions to defer further prednisone and follow up with outpatient dermatology, nephrology, and rheumatology.

This patient’s presentation with a widespread rash, systemic symptoms, and recent immunosuppressive therapy prompted a broad differential diagnosis. Initial considerations included infectious etiologies, possibly due to Parainfluenza virus versus other causes of pneumonia; inflammatory causes related to underlying FSGS; or autoimmune due to underlying vasculitis, such as IgA vasculitis, polyarteritis nodosa, and microscopic polyangiitis. Drug reaction with eosinophilia and systemic symptoms (DRESS syndrome) was also considered, given the combination of rash, pulmonary infiltrates, transaminitis, and recent immunocompromised status. However, the absence of high-grade eosinophilia, atypical lymphocytosis, and lymphadenopathy made DRESS less likely. ANCA-associated vasculitides remained on the differential diagnosis due to pulmonary and renal involvement but were unsupported by serologic testing and imaging. IgA vasculitis was also considered but was less favored given the patient’s age and absence of gastrointestinal involvement. Other infectious causes, including viral exanthems, fungal pneumonia, and disseminated bacterial infections, were ruled out through molecular testing and bronchoscopy.

Ultimately, the diagnosis of Parainfluenza-associated leukocytoclastic vasculitis was supported by biopsy findings of small-vessel vasculitis with prominent eosinophilia and the temporal association with recent Parainfluenza infection.

## Discussion

LCV is a complex condition that may require a multidisciplinary approach to diagnose and manage. This case highlights a broad list of differential diagnoses, particularly in patients of darker skin tones with atypical presentations or involving mucosal surfaces and multi-organ involvement. Pigmentary changes in darker skin may cause LCV lesions to appear differently, potentially masking classic erythema and purpura, leading to underrecognition or delayed diagnosis. Literature on LCV in skin of color is limited, but recent reviews underscore the need for improved dermatologic training and inclusion of diverse skin tones in educational resources. Increasing awareness of these differences is essential to reduce disparities in dermatologic diagnosis and care [7,8].

Our literature review yielded no similar case reports or distinct guidelines on the work-up or management of LCV specifically. When suspicious for LCV, skin biopsies remain the cornerstone for confirmation, with direct immunofluorescence recommended as a helpful tool that can yield diagnostic information about underlying disease pathophysiology [1,9]. Although LCV is often idiopathic, a thorough evaluation guided by clinical signs and symptoms is warranted. Some diagnostic methods include blood count, biochemistry profiles with liver and renal function tests, autoantibody screening, urinalysis, and infectious serologies, which can be necessary to exclude other potential etiologies [4,10]. Given the broad and overlapping clinical features of small-vessel vasculitides and drug reactions, a structured comparison of key characteristics, such as biopsy findings, systemic involvement, and laboratory features, can aid in distinguishing LCV from other mimickers (Table 2).

**Table 2:** Comparison of clinical, histopathologic, and laboratory features of leukocytoclastic vasculitis and commonly overlapping vasculitic or drug-induced conditions. Abbreviations: ANCA = anti-neutrophil cytoplasmic antibodies; c-ANCA = cytoplasmic ANCA; DIF = direct immunofluorescence; ENT = ear, nose, throat; GI = gastrointestinal; IgA = immunoglobulin A; MPO = myeloperoxidase; p-ANCA = perinuclear ANCA; PR3 = proteinase 3; PTU = propylthiouracil

Characteristics	LCV	IgA Vasculitis	ANCA-Associated Vasculitis	DRESS Syndrome		
Typical rash presentation	Palpable purpura, petechiae, often on the lower extremities	Palpable purpura, often on the lower extremities	Petechiae, purpura, nodules, ulcers	Morbilliform rash, facial edema		
Age group	All ages	Children > adults	Middle-aged to elderly	All ages, more common in adults		
Systemic involvement	Rarely systemic	Common (GI, renal)	Common (renal, pulmonary, ENT)	Common (hepatic, renal, pulmonary, hematologic)		
Biopsy features	Neutrophilic infiltration, fibrinoid necrosis	IgA deposition in vessel walls (on DIF)	Granulomatous inflammation, necrotizing vasculitis, fibrinoid necrosis	Eosinophilic and lymphocytic infiltration, interface dermatitis, and perivascular inflammation		
Lab features	None specific	Elevated serum IgA, hematuria	Positive ANCA (c-ANCA (PR3), p-ANCA (MPO))	Eosinophilia, atypical lymphocytes		
Common triggers	Infections, drugs, and autoimmune diseases	Infections, vaccinations	Autoimmune, occasionally drug-induced (e.g., hydralazine, PTU)	Drugs (anticonvulsants, antibiotics)		
Key References	[11,12,13]	[14,15]	[13,16]	[13,17]		

For limited LCV, treatment involves elimination of the causal agent, if known, and symptomatic relief with rest, nonsteroidal anti-inflammatory drugs (NSAIDs), and low-dose topical steroids. When there are extra-cutaneous manifestations of LCV refractory to initial treatments, escalation to systemic corticosteroids, dapsone, colchicine, and other immunosuppressive agents may be required, along with inter-disciplinary collaboration [9]. Most cases of LCV resolve within weeks to months, but in patients with systemic organ involvement, preventing disease progression and minimizing organ damage is crucial.

## Conclusions

This case underscores the importance of early recognition and comprehensive evaluation in patients with LCV, particularly when systemic involvement is present. Our patient's presentation with multi-organ dysfunction highlights the need for a broad differential diagnosis and timely clinical decision-making. His favorable outcome reinforces the effectiveness of prompt, multidisciplinary care in managing complex LCV presentations. Importantly, this case highlights diagnostic challenges associated with LCV in patients with darker skin tones, where erythema and purpura may appear less conspicuous. Expanding dermatologic education and improving representation of skin of color in clinical materials are essential steps toward reducing diagnostic delays and advancing health equity in dermatologic care.

A structured diagnostic approach, guided by clinical presentation, laboratory findings, and histopathologic confirmation, is critical in distinguishing LCV from mimicking conditions. While most cases resolve with appropriate management, patients with complex or refractory disease benefit from additional education, close follow-up, and regular monitoring to prevent recurrence or complications. This case contributes to a growing body of literature emphasizing the multisystem potential of LCV and the diagnostic nuances in richly pigmented skin. Future research exploring phenotype-specific presentations, diagnostic tools, and treatment outcomes across diverse populations could inform more inclusive and equitable clinical guidelines.
